# Miniaturized Raman Instruments for SERS-Based Point-of-Care Testing on Respiratory Viruses

**DOI:** 10.3390/bios12080590

**Published:** 2022-08-02

**Authors:** Ahmed Ali, Ezekiel Edward Nettey-Oppong, Elijah Effah, Chan Yeong Yu, Riaz Muhammad, Toufique Ahmed Soomro, Kyung Min Byun, Seung Ho Choi

**Affiliations:** 1 Department of Electrical Engineering, Sukkur IBA University, Sukkur 65200, Pakistan; ahmedali.shah@iba-suk.edu.pk; 2 Department of Biomedical Engineering, Yonsei University, Wonju 26493, Korea; ezekieledward@yonsei.ac.kr (E.E.N.-O.); myelijaheffah@yonsei.ac.kr (E.E.); yu01010356@yonsei.ac.kr (C.Y.Y.); riaz.be17@iba-suk.edu.pk (R.M.); 3 Department of Electronic Engineering, Quid-e-Awam University of Engineering, Science and Technology, Larkana 77150, Pakistan; toufique_soomro@quest.edu.pk; 4 Department of Biomedical Engineering, Kyung Hee University, Yongin 17104, Korea; 5 Department of Electronics and Information Convergence Engineering, Kyung Hee University, Yongin 17104, Korea; 6 Department of Integrative Medicine, Major in Digital Healthcare, Yonsei University College of Medicine, Seoul 06229, Korea

**Keywords:** respiratory viruses, pandemic, point-of-care testing device, Raman scattering, surface-enhanced Raman spectroscopy

## Abstract

As surface-enhanced Raman scattering (SERS) has been used to diagnose several respiratory viruses (e.g., influenza A virus subtypes such as H1N1 and the new coronavirus SARS-CoV-2), SERS is gaining popularity as a method for diagnosing viruses at the point-of-care. Although the prior and quick diagnosis of respiratory viruses is critical in the outbreak of infectious disease, ELISA, PCR, and RT-PCR have been used to detect respiratory viruses for pandemic control that are limited for point-of-care testing. SERS provides quantitative data with high specificity and sensitivity in a real-time, label-free, and multiplex manner recognizing molecular fingerprints. Recently, the design of Raman spectroscopy system was simplified from a complicated design to a small and easily accessible form that enables point-of-care testing. We review the optical design (e.g., laser wavelength/power and detectors) of commercialized and customized handheld Raman instruments. As respiratory viruses have prominent risk on the pandemic, we review the applications of handheld Raman devices for detecting respiratory viruses. By instrumentation and commercialization advancements, the advent of the portable SERS device creates a fast, accurate, practical, and cost-effective analytical method for virus detection, and would continue to attract more attention in point-of-care testing.

## 1. Introduction

Raman scattering was initially introduced in the early twentieth century when various quantum scientists forecasted it between 1923 and 1927, and then was confirmed experimentally by Indian scientist Chandrasekhara Venkata (C.V) Raman in 1928 [1]. In 1930, Sir C. V. Raman and his team were awarded the Nobel Prize in Physics for discovering this novel type of light scattering [2]. The first experiments were performed on fluids using sunshine as a source of excitation and the bare eyes as a sensor; both the sunshine and the scattering of light were screened to show that the scattered radiation had a longer wavelength than the incident radiation, indicating that Rayleigh scattering really could not have occurred [1]. Since then, instrumentation for recording spectra has improved, benefiting from technological advancements [3]. In the 1960s, the laser was introduced, providing a monochromatic and extremely powerful source of light for analytes’ stimulation [2]. The development of the charge-coupled device (CCD) during 1980, with multichannel architecture and excellent quantum efficiency, significantly increased the signal-to-noise ratio (SNR) [4]. The earliest Raman spectrometers model was developed in 1968, and it featured great imaging capabilities and spatial resolution [5]. Furthermore, during the early nineties, the advent of filters for holographic rejection allowed the Raman experiment to be considerably easier and smaller, restricting the use of triple spectrographs to a few applications [6]. The first commercially viable tabletop Raman machine was produced in 1992. Recently, instrumentation development trends point to device miniaturization in order to make the devices more portable and suitable for point-of-care examination [7]. The widespread commercialization of big products such as compact disk (CD) players and digital cameras greatly facilitated this trend [8]. In fact, the broad use of these electronic devices coincided with advancements in two important elements of a Raman device: the laser and CCD [8]. 

Compact diode lasers, frequently used in CD players, were initially inappropriate for the excitation of Raman spectra because of the abrupt transition from the distinct longitudinal modes roughly 10 cm^−1^ apart synonym, a distinctive characteristic of diode sources [9]. Once this challenge was overcome technically, and the laser emission was fixed to a single mode, diode lasers became perfect sources for Raman instruments. There are now tiny laser instruments with a broad range of emission spectra from near-infrared to visible that are accessible at a reasonable price. Similarly, the introduction of digital cameras to the market aided in the reduction in CCD size and cost [8].

### 1.1. Raman Scattering Principle

**Figure 1:**
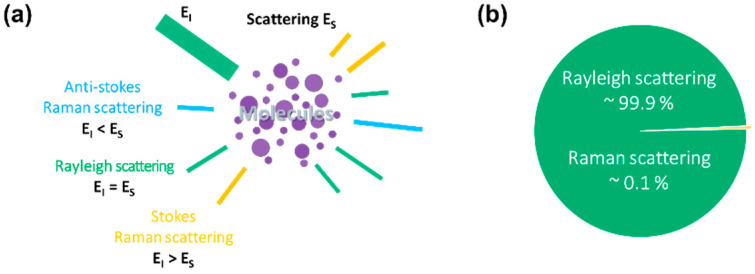
Schematic of Raman scattering. (**a**) Raman scattering is classified into Stokes and anti-Stokes Raman scattering based on the vibrational energy changes. Relative to the incident energy, vibrational energy of the scattered light is lower in the case of Stokes Raman scattering, higher in the case of anti-Stokes Raman scattering, and constant for Rayleigh scattering. (**b**) The scattering of photons by irradiated molecules is predominantly elastic (Rayleigh scattering), accounting for approximately 99.9% of the scattered light. The remaining 0.1% of the scattering is attributed to an inelastic Raman scattering.

The Raman effect is an inelastic scattering of photons characterized by a change in the vibrational energy of molecules upon interaction with light [10,11,12]. The interaction results in molecular energy transitions which further classify Raman scattering into Stokes and anti-Stokes Raman scattering. Relative to the incident energy, vibrational energy becomes lower in the case of Stokes Raman scattering, higher in the case of anti-Stokes Raman scattering, and constant for Rayleigh scattering, as illustrated in Figure 1a.

The atoms of a material are polarized when irradiated by light. This induces an electric dipole since exposure to an electromagnetic field displaces negatively charged subatomic particles from the atomic nuclei. The electric dipole moment induced from irradiation is directly proportional to the strength of the electromagnetic field, where the constant of proportionality denotes the polarizability [12]. Molecular polarizability is the deformation of the electron cloud of a molecule by an external electric field [13]. Polarizability is static for elastic scattering (Rayleigh scattering); hence, the external electromagnetic field and scattered electromagnetic wave have an identical frequency. In the case of inelastic scattering (Raman scattering), polarizability changes with time due to molecular vibrations from electron density changes in response to the nuclear motion [12]. The induced electric dipole moment can be quantified using the Taylor expansion series as:


(1) $$µ=\alpha_{0}E_{0}\;\cos{𝜔}_{0}\;𝑡+\frac{1}{2}\left(\frac{\partial \alpha }{\partial 𝑞}\right){|}_{𝑞=0}\cdot \;{𝑞}_{0}E_{0}\;\cos\;({𝜔}_{0}-{𝜔}_{𝑅})\;𝑡+\frac{1}{2}\left(\frac{\partial \alpha }{\partial q}\right){|}_{𝑞=0}\cdot \;{𝑞}_{0}{𝐸}_{0}\;\cos\;({𝜔}_{𝑅}+{𝜔}_{0})\;𝑡$$

The time-dependent induced electric dipole moment *µ* is evaluated at the nuclear equilibrium position (𝑞 = 0) as a function of polarizability *α* and frequency 𝜔. The equation expresses the radiation scattering by molecules as a superposition of three frequencies at which the induced electric dipole moment oscillates.

In the first term, the oscillation frequency 𝜔_0_ is identical to the frequency of the external electromagnetic field, representing Rayleigh scattering. The subsequent terms express modulated frequencies of oscillations, thus, the deviation between the frequency of the external electromagnetic field 𝜔_0_ and the vibrational mode frequency 𝜔_𝑅_. The frequency drop in the second term depicts the excitation corresponding to red-shifted inelastic scattering as compared to the incident light. Excitation occurs when molecules make upward energy transitions by taking up energy from the incident electromagnetic field, resulting in a lower frequency (𝜔_0_ − 𝜔_𝑅_). The third term depicts de-excitation due to frequency gain, corresponding to blue-shifted inelastic scattering. De-excitation occurs when molecules make downward energy transitions in anti-Stokes Raman scattering, resulting in a higher frequency (𝜔_0_ + 𝜔_𝑅_) [14]. The second and third terms represent Stokes Raman Scattering and anti-Stokes Raman Scattering, respectively.

Rayleigh scattering is the elastic scattering of photons upon interaction with particles [13]. The elastic scattering forms approximately 99.9% of the total scattered light (Figure 1b). In contrast, inelastic Raman scattering is the minor fraction of the total scattered light, corresponding to the remaining 0.1%.

### 1.2. From Raman to Surface-Enhanced Raman Scattering (SERS)

**Figure 2:**
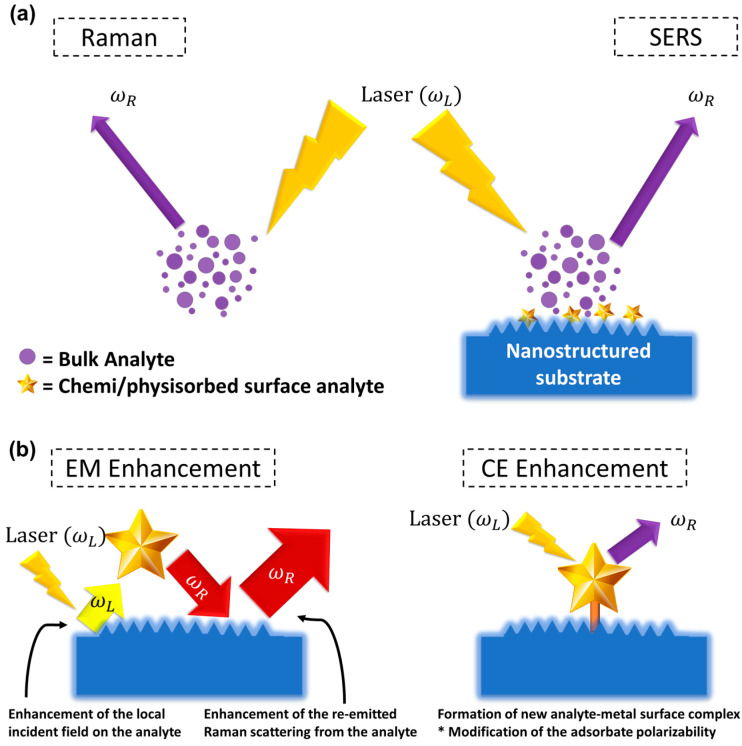
(**a**) A schematic comparison of the Raman and SERS phenomena. (**b**) SERS electromagnetic and chemical enhancements’ schematic.

After several years since Fleischmann et al. [15] discovered an unexpectedly big inelastic scattering from some molecule absorbed on the rough noble metal surface, Van Duyne and Jeanmaire [16] and Creighton and Albrecht [17] separately established that noble metal substrates with nanomaterials’ characteristics are at the root of the significant intensity enhancement. Nevertheless, it should be noted that the SERS process is more than just a Raman spectrum enhancement; it is the result of a number of factors that can affect the intensity of the resulting Raman spectrum [18]. In reality, the average Raman intensity in standard Raman spectroscopy is a linear function of the following factors, excitation wavelength, molecule scattering efficiency, the quantity of sample, and density of laser power. This linear function can only be transformed to SERS if certain criteria are met, and the presence of nanomaterials adds a significantly increased level of complexity to the experiment, which primarily involves the noble metals’ attributes. Furthermore, various additional processes, such as surface complex formation and new surface selection, may also lead to a significant change in the SERS signal in comparison to the initial conventional Raman Signal. Figure 2a provides a schematic representation of the Raman and SERS. Recent studies [18,19] presented a comprehensive review of SERS fundamentals, giving newcomers to the subject a gradual introduction to the method’s intricacy and the techniques needed to manage the more technical aspects [20,21,22,23,24]. 

All elements of SERS have been extensively researched, including the physical mechanisms that govern signal amplification, substrate manufacturing and optimization, materials appropriate for SERS amplification, and applications. SERS applications include a wide range of sectors and are constantly expanding. For example, SERS-based sensing has been used to detect narcotics, pigments, biological species, contaminants, food additives, warfare agents, and explosives.

### 1.3. Enhancement Mechanisms

It is now widely believed that SERS enhancement can be attributed to two synergistic effects of chemical enhancement (CE) and electromagnetic (EM) enhancement.

#### 1.3.1. Chemical Enhancement

The first synergistic component to SERS amplification is based on a variation in the polarizability of the molecule absorbed on the surface of the metal nanomaterial, resulting in the development of new metal-analyte surface interactions [25] (Figure 2b). The resulting change in the electronic configuration of the absorbed molecule may permit for more electronic alterations inside the surface binding, as reported in resonant Raman scattering [26], which causes a parallel rise in the Raman cross-section. As a result, the CE is related to the variation in the polarizability of the absorbed molecule [19,25] rather than the SERS process itself. CE, therefore, is analyte-dependent and, unlike electromagnetic enhancement, contributes far less to overall amplification (usually 10^1^–10^2^) [18].

#### 1.3.2. Electromagnetic Enhancement

The second synergistic component is the electromagnetic enhancement, which may be as high as ~10^10^–10^11^, a major contributor to the large increase in the SERS signal [23]. The presence of EM enhancement is a requirement for observing the SERS effect when localized surface plasmon resonance (LSPRs) is stimulated via the electromagnetic interaction of metals with light, and its origin is due to substantial local field amplifications that pertain to a molecule positioned at or near the nanomaterial surfaces. As a result, the type of electromagnetic amplification is inextricably linked to nanomaterials’ parameters (inherent dielectric, shape, and size, size of the metal nanomaterial) and applies to all analytes [18,19]. To clarify, local field amplification on the metal nanomaterial has two distinct synergistic influences on the Raman signal: the first comes from amplification in the local field on the sample, and the second comes from an increase in the analyte’s re-emitted Raman scattering (Figure 1b). The Electromagnetic-enhancement factor (EF) “realized” by a single molecule positioned at a fixed location on the metal nanomaterial surface at the Raman scattering frequency,$\;\omega_{R}$, excitation frequency,$\;\omega_{L}$, is given by the following formula:

(2) $$EF=\frac{{\left|E_{Loc}\left(\omega_{L}\right)E_{Loc}\left(\omega_{R}\right)\right|}^{2}}{{\left|E_{Inc}\right|}^{4}}\;\approx \;\frac{{\left|E_{Loc}\left(\omega_{L}\right)\right|}^{4}}{{\left|E_{Inc}\right|}^{4}}$$

where $E_{Loc}$ is the local field on the sample; $E_{Inc}$ is the incident field on the metal nanomaterial [27]. If the metal nanomaterials are assumed to be spherical, the local field enhancement of a sphere of radius *a* diminishes rapidly as the separation *d* from the surface increases [19], thereby decaying at 1/(*d* + *a*).

Furthermore, the intensities of the normal and tangential elements of the local field differ, and their relative ratio changes with frequency. When compared to standard Raman settings, this leads to a unique collection of surface selection criteria for a Raman scatterer with a definite orientation at the metal surface, which might lead to the development of different Raman modes and alterations in the intensity of the wavebands [18,25,28,29]. Despite recent interest in single-molecule SERS sensing [19,30], the most meaningful SERS uses rely on acquiring an averaged spectrum intensity from a reasonably large amount of scatterers at the metal nanomaterial surface. As a consequence, the mean SERS mode has better signal repeatability and stability, making it suitable for precise quantitative analysis, while weaker enhancement factors (usually in the range of 10^5^–10^7^) are produced, as compared to those recorded for single molecules’ detection positioned at the hotspots of extremely powerful SERS metal nanomaterials (around 10^10^ or more). Consequently, the average EF of the SERS substrate gives better data for practical SERS applications than the case of finding the local amplification at a certain location on the surface [19]. *EF_avg_* is expressed as:

(3) $$EF_{avg}=\frac{I_{SERS}/N_{Surf}}{I_{RS}/N_{Vol}}$$

where *EF*_avg_ is the average enhancement factor; $N_{Surf}$ is the number of molecules absorbed on the metal nanomaterial surface; $N_{Vol}$ is the number of molecules in the scattering volume; $I_{RS}$ is the intensity of the Raman signal, and $I_{SERS}$ is the intensity of the SERS signal. It should be noted that the *EF*_avg_ depends on several factors that include characteristic of the metallic nanostructure, the concentration of the Raman reporter on the metallic nanostructure, and the frequency of the excitation beam. Thus, it is difficult consistently to calculate and compare the *EF*_avg_ published in different literatures [19].

Overall, (i) the systematic discovery of SERS, which has dramatically increased sensitivity, and (ii) technological advancements that have produced compact, very sensitive, and simple-to-use Raman devices are both benefiting the spread of Raman spectroscopy across disciplines and its transformation from a scientifically difficult study technique to a more commonly accessible analysis method.

**Figure 3:**
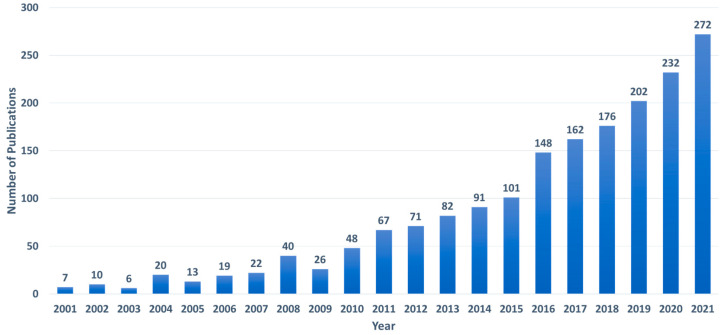
The number of articles published each year employs a handheld Raman equipment to identify the substance.

We performed a bibliographic search to identify current dissemination research reports involving SERS detection of analytes using portable Raman devices. The following strings were entered into the Web of Science abstract, field: “(Handheld OR Portable) AND (Surface enhanced Raman OR SERS)”. Furthermore, the language was limited to English, and all types of papers, including conference proceedings, were accepted. We excluded the papers that were irrelevant to our search (for example, some referred to handheld SERS substrates rather than handheld devices). Figure 3 shows a summary of the findings that the number of articles published using handheld Raman devices for SERS sensing has been increasing steadily over the last 5–10 years. Despite the fact that there have been several publications and reviews published in recent years on food, pharmaceuticals and medicine, explosives and warfare, and the environment, no study has been expressly devoted to the application of handheld devices for the sensing of virus molecules. The goal of this work is to close this space by concentrating on the sensing of viruses using a portable Raman device. It is worth noting that, throughout this review, we will refer to portable instruments as a whole, regardless of whether they are small enough to be termed handheld or just transportable. Our review is organized as follows: In Section 2, we introduce the Handheld Raman spectrometers. In Section 3, we compare bench-top and portable Raman spectrometers. In Section 4, we introduce SERS substrates for the point-of-care diagnosis. In Section 5, we introduce the state-of-the-art SERS-based diagnosis of viruses.

### 1.4. Factors to Consider for SERS Measurement at the Point of Care

**Figure 4:**
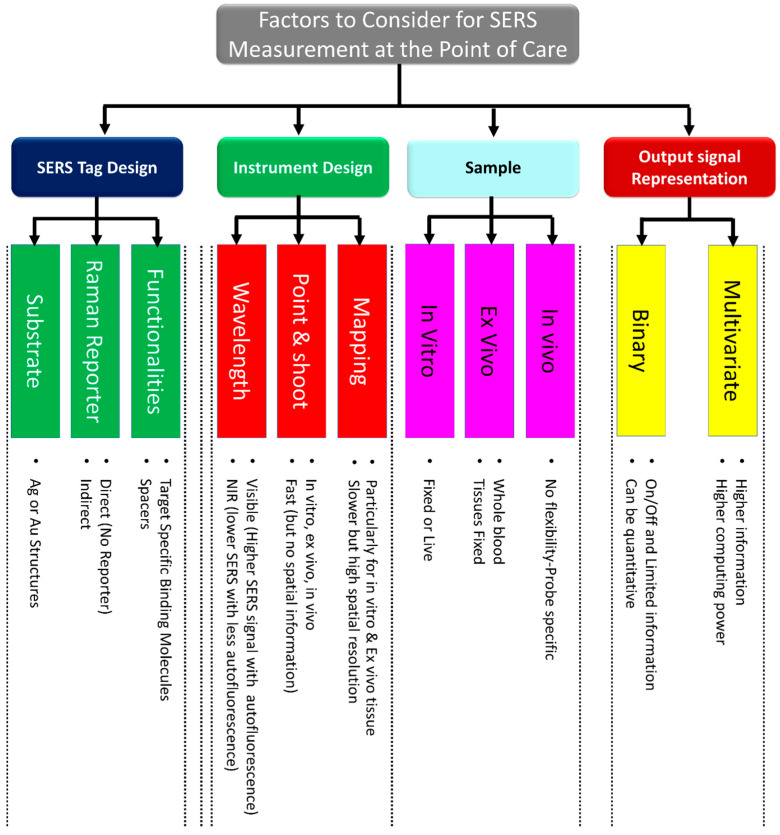
List of the most important factors to consider when performing SERS measurements at the point of care.

This section highlights the critical factors to consider when performing analytical measures at the point of care. To perform optimal measurements for specific applications at the point of care, a number of factors must be carefully considered, including the SERS probe, instrument, sample preparation, and output signal representation. A summary of these factors is depicted in Figure 4.

#### 1.4.1. SERS Probe Choice

When choosing the optimal SERS probe for a specific point-of-care measurement, several factors must be considered. Metal nanoparticles or substrates modified with a targeting molecule and Raman dye are important parts of the SERS probe. Because of their excellent optical properties, Au and Ag nanostructures are commonly used for the SERS probe. These two metal nanostructures exhibit surface plasmon resonance (SPR) in the near-infrared and visible ranges. The SPR of these nanostructures may be controlled by carefully selecting the nanostructure dimension morphology and shape. By choosing a nanostructure with the appropriate SPR value, the best excitation wavelength for a specific study can be employed. Au [31] and functionalized Ag [32] nanostructures are also non-toxic when used in biological systems.

Particularly, Au nanostructures are non-toxic and have been authorized for use in live subjects for specific studies [33]. As a result, Au nanostructures are frequently used as the SERS nanoprobe for in vivo and in vitro studies. Ag nanostructures, however, exhibit better scattering characteristics and greater Raman signal amplification [34]. Ag-nanostructures-based SERS probes may thus be preferred in ex vivo studies when analytes are not being administered directly to live subjects. While raw Ag nanostructures are proven to be cytotoxic, raw Ag is therefore not preferred for in vivo and in vitro applications. However, researchers reported that this toxicity may be reduced following Ag nanostructures’ surface modifications. The mechanism underlying Ag nanostructure toxicity and its reduction following surface modification are unknown; however, they are attributed to factors such as the biological medium, coating agents, charge, shape, size, and surface area [35]. Biomolecules and Raman labels can be covalently or electrostatically coupled to the SERS probe [36]. Capping agents, including SiO_2_ and Polyethylene glycol (PEG), are frequently used to functionalize nanostructures for a variety of reasons, including preventing dissolution, reducing cytotoxicity, and facilitating further modifications [36]. Finally, bio-recognition agents can be added to the surface of the SERS probe to target a specific molecule. These agents include antibodies and oligonucleotides that target particular proteins and RNA/DNA [37]. There are several methods for attaching dyes and biomolecules to SERS probes, including covalent bonding via, for instance, EDC-NHS attachment [38] or electrostatics interactions between the dye and the SERS probe surface [39].

#### 1.4.2. Instrument Choice 

The design of the Raman spectrometer is another crucial factor to take into account when employing SERS measurement at the point of care. The best instrument for a specific measurement is selected by considering the type and amount of data required, the sample, and the SERS probe. The choice of the SERS probe, however, may make this selection more challenging since the laser excitation wavelength that would produce the best performance can differ depending on the contributions from the Raman reporter dyes and the nanostructures’ plasmon resonance. This is further complicated by the presence of background fluorescence from the bio environment, as well as the low depth of tissue penetration of visible excitation wavelengths. As a result, many Raman recordings’ experiments are currently being conducted using infrared lasers [40,41] that can aid in reducing background fluorescence and improving tissue penetration depth. The size of the Raman instrument for point-of-care measurement should be such that it can fit in a typical human hand. The point-and-shoot Raman measurement style can be used for in vivo, ex vivo, and in vitro recordings at the point of care. It is ideal for point-and-shoot measurements to identify chemicals quickly at the touch of a button. The user of the Raman instrument can simply identify unknown samples such as virus chemical warfare agents or verify incoming goods without being a scientifically trained person. The Raman instrument should also have simple-to-use software and accessories for assessing the hazards associated with substances and making life-saving decisions on the spot or in high-risk circumstances. 

Studies requiring spatial data about the distribution or placement of a biomarker or tag, which is common in ex vivo and in vitro research, are frequently dependent on SERS measurements based on confocal mapping in 2D or 3D [42]. In vivo mapping has also recently was demonstrated in studies [43]. However, spatial data Raman measurements are not appropriate for point-of-care diagnostics.

#### 1.4.3. Sample Preparation

The preparation of a sample for SERS measurement at the point of care must be carefully considered by researchers. The first and most important step in diagnostics at the point of care is the capacity to identify and characterize the analyte of interest from small sample volumes quickly and accurately. In a perfect SERS test, sample preparation would be simple and inexpensive; several serial samples could be automatically analyzed, and analytes could be quickly classified using a reliable database.

Direct and indirect SERS sensing are two different ways to measure SERS. In direct SERS sensing, samples are mixed or directly deposited on a SERS active surface, allowing for the detection of the SERS spectrum from its constituent parts. Principal component analysis (PCA) or other sophisticated machine learning techniques are frequently needed for the examination data. This approach is simple to use and often involves little sample preparation. It is more difficult to convince medical experts and regulatory bodies to approve the test due to the intricacy of the signal, which makes it difficult to determine which sample elements are responsible for the diagnostic result. On the other hand, for indirect SERS detection, Au and Ag nanostructures can be utilized as SERS nanoprobes. The Raman reporter molecule with the known signal is coated on Au/Ag nanostructures to create SERS nanoprobes. This technique can make use of processes comparable to existing conventional practices, including lateral flow, ELISA, or PCR tests, but with a transduction based on SERS. Due to this characteristic, indirect SERS detection is more likely to be used in clinical settings, and efforts are now being made to translate these techniques from the laboratory to the point of care.

The specimen can be fixed or live in studies requiring in vitro samples. Recording data are frequently only meaningful when made with living specimens, as in pH sensing with SERS probes [44]. Fixed specimens are much simpler to manage when Raman probes are used because specimen cytotoxicity could be reduced. On the other hand, the process of fixing the specimen may produce artifacts and chemically alters them. Artifacts might include physical shrinkage of the specimen, deposition of fixing molecules or other reagents used in the fixation procedure, or even the inherently chemical alteration used for fixation. There are no sample preparation stages necessary for in vivo measurements because the patient must remain alive during the procedure; instead, the SERS probes must be created in a way that they can function in a living environment. Researchers must always take into account the possibility of other biomolecules interfering with SERS measurements, such as the presence of extra blood proteins in blood samples and the presence of bovine serum albumin (BSA) in cell culture conditions [45]. Additionally, background fluorescence might interfere with measurements, especially for in vivo samples. However, proper laser wavelength selection can lessen these effects.

#### 1.4.4. Output Signal Representation 

The methodologies for assessing the collected data must be taken into consideration for point-of-care applications. SERS nanoprobes can be employed to provide an on or off response to a coupling event, much like fluorescence. This could be quantified if a SERS band’s magnitude is calibrated using values that are well-known or have been independently observed. This serves as an illustration of a univariate approach. More complex statistical approaches, including multivariate analysis, are frequently used when evaluating SERS data. When SERS probes are used to assess physiological signals directly, the resulting spectra provide information on a range of biomolecules present near the nanoparticle surface. Principle component analysis is frequently used in this situation to minimize signal dimensionality by generating principal components that account for the most variance in the data set [18]. Another method is partial least squares regression analysis, which models the spectrum responses to predetermined incremental experimental adjustments [18]. This has been utilized to increase the capabilities of multiplex SERS tags by enabling more accurate measurement and differentiation of specific tag contributions to multiplex signals [19]. The direct classical least squares method is a multivariate approach used to differentiate contribution from distinct SERS tags in a multiplex signal [20,21]. 

## 2. Handheld Raman Spectrometers 

The Raman spectroscopy setup can be coupled with an optical microscope to provide high spatial resolution on the order of 1 µm. Such customization is termed micro-Raman spectroscopy or Raman microscopy, and it is more commonly used than the Raman spectrometer [46]. Micro-Raman spectrometers typically require additional optical instruments unlike Raman spectrometers, which are relatively simple. Due to technological advancements, conventional Raman spectrometers have been further simplified to develop handheld Raman spectrometers (Table 1).

Handheld Raman spectrometers have a significant advantage over other characterization techniques since they do not require the pretreatment of samples or direct contact with samples. In field applications, these portable Raman devices can analyze samples through transparent containers such as plastic and glass. Handheld Raman spectrometers are efficient in material identification [47,48,49], authentication of finished products for quality control [50,51,52,53], and anti-counterfeit prevention [54,55,56,57].

A typical handheld Raman spectrometer such as the I-Raman PLUS from B&W Tek has a high quantum efficiency CCD array detector with a high dynamic range and deeper cooling. This handheld Raman spectrometer provides an improved signal-to-noise ratio for up to 30 min, making it feasible to measure weak Raman signals [58]. The commercialization of handheld Raman spectrometers has created an avenue for point-of-care diagnosis [59,60]. It eliminates the time-consuming process of sending samples to a laboratory for testing.

The portability and simplified user interface software of handheld Raman spectrometers have expanded their application in various industries, including the military. Users do not need any prior understanding of analytical chemistry to operate these spectrometers. In addition, it is an efficient diagnostic tool for viral infections in plants. Handheld Raman spectrometers have been used in the early detection of *Abutilon mosaic virus* (AbMV) (family *Geminiviridae*; genus *Begomovirus*) [61], an important virus that infects ornamental plants all over the world.

Handheld Raman spectrometers are more resilient than bench-top spectrometers, despite their small size and light weight. This is because handheld Raman spectrometers are frequently used in field applications and are more likely to be dropped or involved in other accidents. Furthermore, handheld Raman spectrometers are designed to consume less power to enable longer operation periods between recharges.

**Table 1:** Laser, detector, and configuration of handheld Raman spectrometers. Handheld Raman spectrometers. Commercially available Raman spectrometers from Thermo Fisher Scientific (Waltham, Massachusetts, USA) [62], Rigaku (Tokyo, Japan) [63], Metrohm (Herisau, Switzerland) [64], B&W Tek (Newark, DE, USA) [65], B&W Tek (Newark, DE, USA) [66], Thermo Fisher Scientific (Waltham, Massachusetts, USA) [67], Rigaku (Tokyo, Japan) [68], Bruker (Billerica, Massachusetts, USA) [69], B&W Tek (Newark, DE, USA) [70], B&W Tek (Newark, DE, USA) [58], and Ocean Insights (Orlando, FL, USA) [71]. Owing to the extraordinarily high molecular selectivity of the Raman technique, these handy spectrometers are ideal for in situ analysis of illicit narcotics, raw material testing, final product verification, quality control, and counterfeit drug detection, among other chemical and pharmaceutical applications.

Portable Raman Spectrometers	Laser Wavelength and Power	Raman Spectroscopy Geometry	Detector
Thermo Fisher Scientific, Gemini [62]	783 nm	Backscattered geometry	N/A
Rigaku, Progen [63]	1064 nm, 30–490 mW	Backscattered geometry	TE cooled InGaAS photodiode
Metrohm, Mira XTR DS [64]	785 nm, 100 mW	Backscattered geometry	NIR enhanced back thinned CCD
B&W Tek, TacticID GP Plus [65]	785 nm, 30–300 mW	Backscattered geometry	CCD array
B&W Tek, NanoRam [66]	785 nm, 30–300 mW	Backscattered geometry	TE-cooled CCD array
Thermo Fisher Scientific, TruScan RM [67]	785 nm, 250 mW	Backscattered geometry	N/A
Rigaku, Progeny ResQ [68]	1064 nm, 30–490 mW	Backscattered geometry	TE cooled InGaAS photodiode
Bruker, Bravo [69]	700–1100 nm (Duo LASER^TM^), <100 mW	Backscattered geometry	CCD array
B&W Tek, QTRam [70]	785 nm, 420 mW	Backscattered geometry	CCD array
B&W Tek. I-Raman Plus [58]	532 nm, 42 mW 785 nm, 455 mW	Backscattered geometry	High quantum efficiency CCD Array
Ocean Insights, QE Pro [71]	785 nm	Backscattered geometry	Back-thinned FFT-CCD detector
Emmanuel et al. [72]	532 nm, 780 mW	Backscattered geometry	Science-Surplus spectrometer, linear CCD detector array (Sony ILX511)
Dhankhar et al. [73]	532 nm, 50 mW	Right angle geometry	Google Pixel camera, CMOS BSI sensor (Sony IMX363 Exmor RS, Sony IMX378 Exmor RS)
Aydogan and Tasal. [74]	532 nm, 150 mW	Backscattered geometry	CCD array (TCD1304DG, Toshiba (Minato, Tokyo, Japan))
Fitzwater et al. [75]	632.8 nm, 0.5 mW	Right angle geometry	GaAs PMT
Bandyopadhyay et al. [76]	514.5 nm, 4 W	Backscattered geometry	PMT (R928, Hamamatsu (Hamamatsu-city, Japan))
DeGraff et al. [77]	532 nm, 10 mW	Right angle geometry	Ocean optics S2000, CCD array (Sony ILX511)
Young et al. [78]	532 nm, 4 mW	Right angle geometry	Ocean optics S2000, CCD array (Sony ILX511)
Mohr et al. [79]	532 nm, 4 mW	Backscattered geometry	Ocean optics USB 4000, 3648-element CCD array (Toshiba TCD1304AP)
Somerville et al. [80]	532 nm, 5 mW	Right angle geometryBackscattered geometry	Ocean optics HR4000, linear silicon CCD array
Montoya et al. [81]	532 nm, 100 mW	Transmission geometry	Canon EOS 70D APS-C (22.5 mm × 15 mm) CMOS

### 2.1. Handheld Raman Device Configuration and Filters

In Raman spectroscopy, a monochromatic laser beam is focused on a material to produce a scattered light. The interaction causes the energy of re-emitted photons to shift up or down relative to the frequency of the incident laser [82]. 

**Figure 5:**
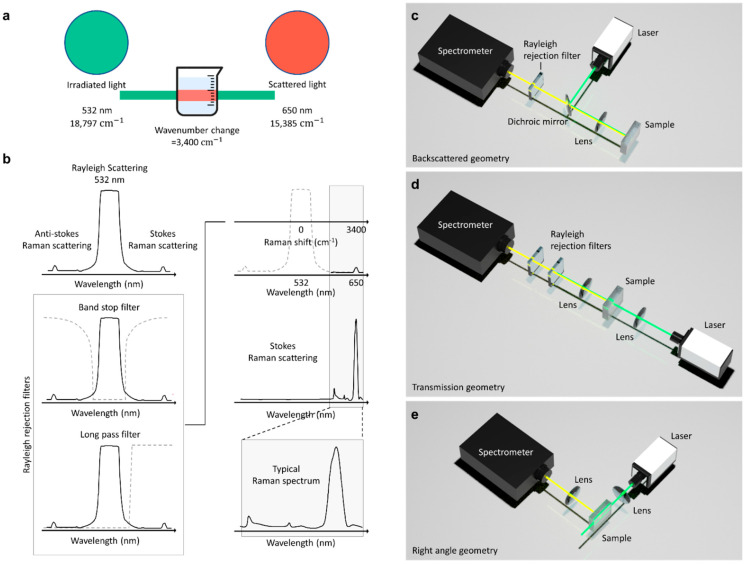
Features of Raman spectroscopy. (**a**) Schematic representation of the Raman shift from light scattering. It can be observed that an incident monochromatic laser with a wavenumber of 18,797 cm^−1^ is scattered at a wavenumber of 15,385 cm^−1^. The resulting wavenumber change of 3400 cm^−1^ is due to the vibrational energy changes of the molecules. (**b**) Effect of long-pass and band-pass filters on generated signals. Recorded signals from light scattering are generated by anti-Stokes Raman scattering, Stokes Raman scattering, and Rayleigh scattering. Rejection filters are required to eliminate Rayleigh signals. They work by allowing and attenuating signals at specified frequencies to measure Raman spectra. Collection geometries in Raman spectroscopy: (**c**) Backscattered geometry, (**d**) Transmission geometry, and (**e**) Right-angle geometry. Backscattered and Transmission geometries require Rayleigh rejection filters to eliminate the noise resulting from Rayleigh scattering and back-reflected excitation light. Unlike the former two geometries, the configuration of right-angle geometry yields little noise, and, hence, rejection filters are not required.

The vibrational energy change associated with the scattering process is expressed as wavenumbers; thus, a change in the wavenumber is evaluated to quantify the scattering. As illustrated in Figure 5a, an incident monochromatic laser with a wavelength of 532 nm and a wavenumber of 18,797 cm^−1^ is scattered at a wavelength and wavenumber of 650 nm and 15,385 cm^−1^, respectively. The observed wavenumber change of 3400 cm^−1^ is known as the Raman shift and informs on the rotational, vibrational, and other low-frequency transitions in molecules.

The recorded spectrum from light scattering has three major signals corresponding to anti-Stokes Raman scattering, Stokes Raman scattering, and Rayleigh scattering. Rayleigh scattering has the highest signal intensity (Figure 5b), since elastic scattering forms a predominant fraction of the total scattered light. Therefore, to obtain a good Raman spectrum for analysis, Rayleigh rejection filters are required to eliminate the Rayleigh signal. The rejection filters are primarily long-pass and band-stop filters. A long-pass filter blocks Rayleigh signals by allowing signals with frequencies lower than a specified cutoff frequency and attenuating signals with frequencies higher than the cutoff frequency. A band-stop filter blocks Rayleigh signals by allowing most signals without alteration and attenuating signals within specific frequency range (i.e., the stop band) to extremely low levels [83].

The frequency-response required of these filters is determined by the configuration used in the Raman spectroscopy setup. Figure 5c–e shows a schematic depicting how these filters are employed and used in Raman spectroscopy. The backscattered geometry (Figure 5c), which is mostly used in commercial Raman spectrometers, yields intense Raman spectra. However, the noise resulting from Rayleigh scattering and back-reflected excitation light is prominent in backscattered geometry. Subsequently, Rayleigh rejection filters and dichroic mirrors are required to measure Raman spectra. In the transmission geometry (Figure 5d), dichroic mirrors are not required because transmission light directly enters the spectrometer. However, the signal from this Raman collection geometry is masked by the intense incident laser line [73]. Hence, highly efficient laser cleanup filters and laser line rejection filters are required to measure Raman spectra. In the right-angle geometry (Figure 5e), the excitation is applied at 90°. Consequently, minimal noise is produced, and rejection filters are not needed. The scattered light collection optics in right-angle geometry collects light from a large area. Therefore, it is easier to measure bulk properties such as the bulk chemical composition of a turbid media in right-angle Raman collection geometry [73].

### 2.2. Cost-Effective Handheld Raman Spectrometers

**Figure 6:**
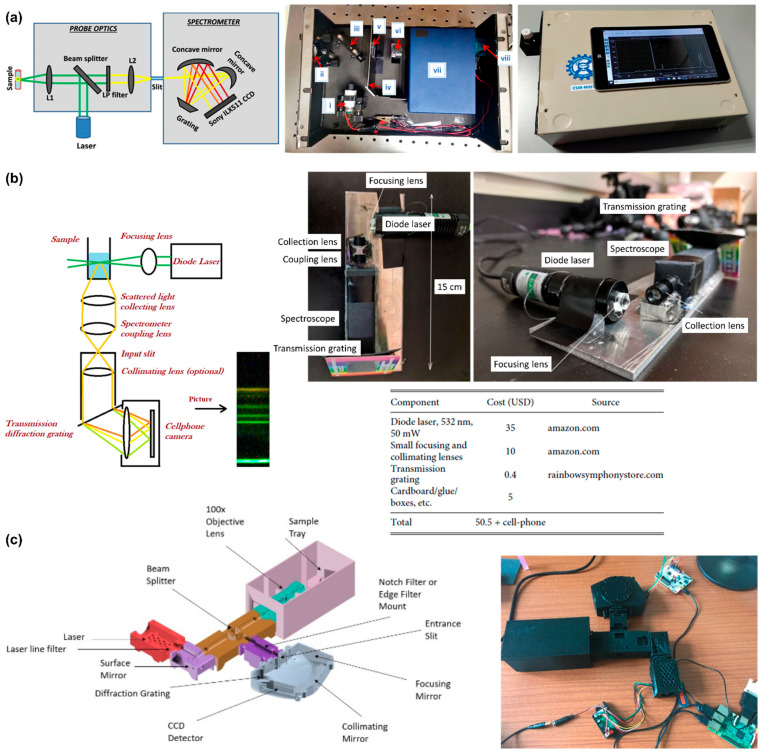
Portable cost-effective Raman spectrometer models. (**a**) Schematic representation of an inexpensive Raman spectrometer (**left**), Components (**middle**): (i) Laser diode, (ii) Focusing lens, (iii) Beam splitter, (iv) Beam block; to prevent interference from the reflected laser and room light, (v) Long-pass filter, (vi) Focusing lens, (vii) Spectrometer, and (viii) RS232 to USB for GUI device, Exterior view (**right**). Reproduced with permission from ref. [72]. Copyright 2021 American Chemical Society. (**b**) Cellphone-based Raman spectrometer constructed from a diffraction grating and cellphone-camera system for in situ detection. Schematic of the spectrometer setup (**left**), top and front view of the system with the parts labeled (**right**), and the expenditure of the components and materials used (**below**). Reproduced with permission from ref. [73]. Copyright 2021 American Institute of Physics (AIP). (**c**) A low-cost, easy-to-handle, Raman spectrometer built with commercial electronics and optics, and 3D printing. A computer-aided design of the 3D printed Raman Spectrometer (**left**) and the corresponding setup (**right**). Reproduced under the Creative Common Attribution 4.0 License (CC BY 4.0) from ref. [74]. Copyright 2018, The Authors, published by CERN.

A major advantage of Raman spectrometry is the simplicity of its setup. Commercial Raman spectrometers are expensive, but, with the same scattering principle, Raman spectrometers can be developed in cost-effective ways. Figure 6 illustrates some of the inexpensive ways of building a Raman spectrometer.

Figure 6a shows the optical layout of a low-cost Raman spectrometer with backscattered geometry. The Raman spectrometer was built using a laser pointer, beam splitter, long-pass filter, two lenses, and a commercial spectrometer [72]. The Raman spectrum was presented on a digital tablet with a Windows operating system, and the power was supplied by a power bank. The setup uses low-cost components to make it simple to construct. The design technique offers the flexibility of configuring Raman spectrometers at various excitation wavelengths, with the only adjustment being the selection of a long-pass filter with the appropriate cutoff wavelength. 

Another way to build a small-budget Raman spectrometer is to employ the camera system of a cell phone [73]. Figure 6b depicts a Raman spectrometer that utilizes a cell phone CMOS camera to record the Raman spectra. The setup is based on the right-angle geometry, and hence no Rayleigh rejection filters are required. The total cost of optical components and accessories required to build the cellphone-based Raman spectrometer is about USD 50.50 excluding the price of cell phone. The device can provide a simple, reliable, and inexpensive method for recording Raman, enhanced Raman, and other optical spectra, such as fluorescence.

The use of 3D printers is another viable way to build a low-cost Raman spectrometer [74]. Figure 6c illustrates a computer-aided design of a Raman setup and an assembled 3D-printed Raman spectrometer. The light collection geometry used in the setup is the backscattered scheme. With commercially accessible electronics and optics, 3D printing offers a cost-effective way to manufacture a Raman spectrometer. Continuous study into the development of affordable and effective Raman spectrometers has the potential to create novel uses of this technology in the daily lives of people.

## 3. A Comparative Assessment of Bench-Top and Portable Raman Device 

It is useful briefly to compare the performance of bench-top and portable Raman devices. Table 2 [84] compares the advantages, disadvantages, and key device parameters of Thermo Scientific Company’s bench-top and portable Raman devices. The Raman signal was measured using the same substrate and sample with both the bench-top and portable Raman devices. It is critical to illustrate the following parameters, as shown in the table.

**Signal Variation** on the analyte is smaller with a portable device due to the device’s significantly larger laser spot, which is linked to a low resolution. A similar observation was made in the study [85] based on the Raman recording of bacteria-labeled SERS probes. It is worth noting that by adjusting the line focus configurations, the bench-top device can also generate a larger illumination [86].

**Sensitivity** is the ability of the SERS probe to detect tiny levels of an analyte in a sample. It is challenging to characterize the sensitivity of SERS measurements because the sensitivity of SERS depends on the excitation power/wavelength, the analyte’s excitation cross-section, and the measurement’s acquisition time. To define sensitivity, the term “limit of detection” (LoD) is frequently used. LoD is the amount of an analyte that could be reliably sensed. To obtain LoD, a sample is diluted until the SERS probe can no longer reliably identify the target in question.

As shown in Table 2, the bench-top device has higher sensitivity and a much lower detection limit (LoD). Zheng et al. [84] found that the intensity of Raman peaks was 20 times greater when using bench-top instruments. Sensitivity is a complicated function of multiple device factors, including the numerical aperture of the spectrometer, the transmittance or reflectivity of the optical components, and the detector efficiency [85]. While a larger numerical aperture enables far more scattered radiation from the sample to be collected, it also requires large optical components in the device to focus the beam from the entry slit to the detector, resulting in a bulkier device. Furthermore, an efficient cooling system can significantly reduce the noise of a CCD detector, thereby increasing the signal-to-noise ratio (SNR) value of spectral measurements at the expense of the Raman device’s size. As a result, a trade-off between instrument performance and size must be made.

**Adjustability.** Portable devices also allow spectral recording on obscuring surfaces, such as pharmaceutical products inside plastic bottles and explosives inside containers, reducing the surface’s contribution. Bench-top devices, on the other hand, reduce the undesirable influence of fluorescence by merging Raman spectra collected at two different excitation wavelengths.

**Excitation Wavelengths.** Portable devices can have excitation wavelengths ranging from near-infrared to visible. Longer wavelength excitation allows noise reduction from fluorescent compounds. The most common wavelength for near-infrared excitation is 785 nm; however, equipment that stimulates further into the red has become widely available. For example, the fluorescence intensity from certain drugs may dominate the Raman spectrum at 785 nm, necessitating the use of 1064 nm excitation sources in pharmaceutical detection.

**Resolution** is smaller in portable devices. Resolution depends on several factors, including excitation wavelength, detector and pixel density, focal length, and slit width. Larger focal lengths and detection devices improve resolution but increase instrument volume. The study by Rasmussen et al. [87] provides a detailed illustration of how a miniaturized Raman device can be developed, as well as a detailed discussion of size constraints. While the common value of resolution ranges from 8 cm^−1^ to 12 cm^−1^ in bench-top devices, the value of resolution in portable devices is much lower, in the order of a few cm^−1^. The Raman bandwidth of liquid and solid materials ranges from 5 cm^−1^ to 10 cm^−1^. Consequently, resolution values from 8 cm^−1^ to 12 cm^−1^ are efficient to differentiate between different chemicals, provided an effective spectral differentiation method is used [8]. 

**Range of Scanning** is also smaller in portable devices. The handheld device described in Table 2 has a scanning range of 250–2875 cm^−1^ and is unable to cover the CH band. The lower scanning range in portable devices is due to the lack of scanning parts and the fixed nature of gratings. The scanning range is usually determined by the manufacturer based on the intended application of the device.

In summary, the bench-top Raman spectrometer has higher sensitivity, higher resolution, and a wider scanning range. Moreover, by modifying device components and parameters such as the objective lens, wattage ratings, and exposure time, strong Raman signals can be recorded with the bench-top device. This means that researchers can obtain the best SERS signals in the lab. However, when compared to the bench-top device, the handheld device demonstrated distinct advantages for SERS sensing, such as mobility, cost-effectiveness, and ease of use. These characteristics make portable Raman devices ideal for point-of-care testing, especially in challenging situations when transportation facilities are unavailable and when samples become chemically unstable over time. Furthermore, a portable Raman device with SERS-based sensing provides a simple and reliable method for detection and quantification; this is far superior to handling measurements manually with a bench-top device using software. Additionally, it is advantageous for individuals who are unfamiliar with Raman measurements or data processing and lack a scientific background.

## 4. SERS Substrates for Diagnosis at the Point-of-Care

The availability of SERS substrates with suitable characteristics is a requirement to spread SERS sensing further with Raman portable devices. SERS substrates should not only be high-performing but also have a consistent reaction throughout the surface and strong repeatability from the substrate to substrate. It is still difficult to find a reasonable balance between performance and repeatability, as both factors are important. Performance affects the sensitivity of the analysis, and repeatability is essential for making quantitative decisions. 

**Figure 7:**
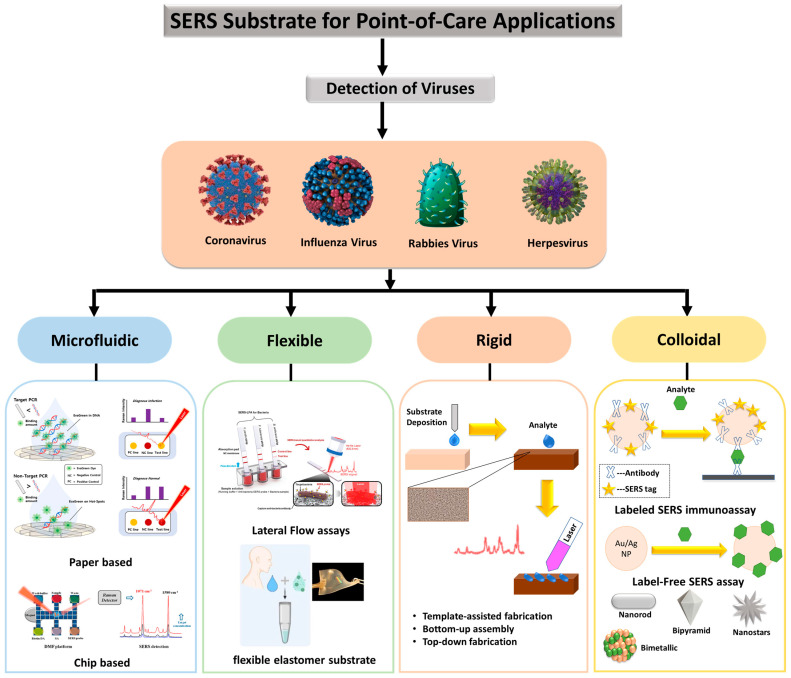
Illustration of many types of point-of-care SERS platforms that have been described, as well as the various types of viruses that have been identified for various applications. Paper-based SERS substrate cartoons reproduced with permission from ref. [93]. Copyright 2021 Elsevier. Chip-based SERS substrate cartoons reproduced with permission from ref. [94]. Copyright 2018 American Chemical Society. Lateral flow assay SERS substrate cartoons reproduced with permission from ref. [95]. Copyright 2016 John Wiley & Sons, Inc. Flexible elastomer substrate cartoons reproduced with permission from ref. [96]. Copyright 2022 American Chemical Society.

Furthermore, scalable manufacturing processes are important for the spread and commercialization of substrates, but difficulties in scalable manufacturing have constrained the widespread use of the SERS platform. Tailoring the substrate properties to meet desired applications is another important feature. Plasmonic structures should be placed on materials with a low Raman signal and should be resistant to the solvents employed to dissolve the test molecule. As depicted in Figure 7, several materials were evaluated for this purpose in recent years, including graphene [88], nanowires [89], silk [90], paper [91], and flexible polymers [92], each of which adds diverse uses to the substrate. These materials enable the creation of SERS substrates that are inexpensive, and expandable for large-scale production. To maintain the excellent specificity of SERS, the molecular interaction affinity of substrates must be considered in the test environment. Aside from these parameters, other requirements of the SERS substrate should be mobility in conjunction with the Raman detector, as well as cost in large-scale production and low-resource circumstances.

For virus detection applications, virus separation mechanisms should be coupled to SERS substrates, allowing one to extract the virus from a complex media and thus facilitate its detection. Recent techniques coupled SERS substrate and separation mechanisms. The coupling is conducted by modifying the SERS substrate with appropriate receptors (e.g., β-cyclodextrines [97], aptamers [98], and antibodies), lateral-flow concentrations (the use of capillary forces in the paper substrate [99]), thin-film chromatography [100,101], and microfluidics [85,102]. Figure 7 depicts several SERS substrates and techniques for virus detection. The use of NIR lasers (1064 nm and higher) is now increased in the production of handheld devices [8,103,104]. As a result, more research and studies are needed to develop the SERS substrate in the NIR spectral range. Above 1064 nm, traditional plasmonic elements such as Ag and Au retain acceptable quality factors for localized SPR [105]. Ag and Au clustering, on the other hand, tends to shift the plasmonic resonance towards the red; these two factors, when combined, create a conducive environment for high sensitivity in this area as well. There are several examples of substrates that have been evaluated at and above 1064 nm in the literature [40,103,106,107,108].

## 5. SERS Based Diagnosis of Virus

SERS detection can be achieved in several ways using both direct and indirect methods [109]. In the direct method, the analytes are detected using their own SERS fingerprints, whereas, in the indirect method, the analytes are detected using the fingerprints of a Raman molecule (also known as reporter, labels, tags, or dyes) embedded in a noble metal nanostructure. The indirect method also necessitates selective binding to the analyte of interest. The direct method is typically utilized to detect small analytes with high Raman cross-sections, such as pesticides, pollutants, and explosives. When dealing with analytes in biological fluids with low Raman cross-sections, such as pathogens, bacteria, and viruses, indirect methods are typically used. The indirect detection method consists of a SERS tag and template: (1) the SERS tag consists of a Raman reporter molecule and a recognition element, which is a particular antibody (detection antibody) localized to SERS-active nanoparticles, and (2) the template, referred to as the capture substrate (not necessarily a metal surface), is functionalized with a linker antibody (capture antibody) to bind the antigen–SERS tag complex. Quantification is conducted by tracking Raman signals from the Raman reporter, before and after the capture element reacts with the SERS tag [110]. Consequently, it overcomes the inherent limitations of using biological analytes in the direct detection method, such as large molecule sizes, low scattering cross-sections, weak affinities for common noble metal SERS substrates, and low specificity [111]. Several reviews for direct detection [112,113,114,115] and indirect detection [116,117,118,119,120] discussed fabrication strategies and their advantages and disadvantages.

### 5.1. SERS-Based Detection of SARS-CoV-2 Virus

Coronaviruses are naturally found in a variety of animal taxa, including birds and mammals (CoVs). However, this type of virus may also infect people, in which case it is referred to as human coronaviruses (HCoVs). In 1966, HCoVs were discovered in the nasal secretions of a rhinitis-infected person in the United States [121]. A novel coronavirus, known as severe acute respiratory syndrome coronavirus 2 (SARS-CoV-2), was recently reported in the city of Wuhan, Wuhan, Hubei Province, China, in December 2019. Commonly referred to as corona virus disease 2019 (COVID-19), SARS-CoV-2 is believed to have possibly originated from a local market for seafood and exotic animal trading [122]. SARS-CoV-2 is the sixth coronavirus to infect humans resulting in high mortality and morbidity worldwide [123].

**Figure 8:**
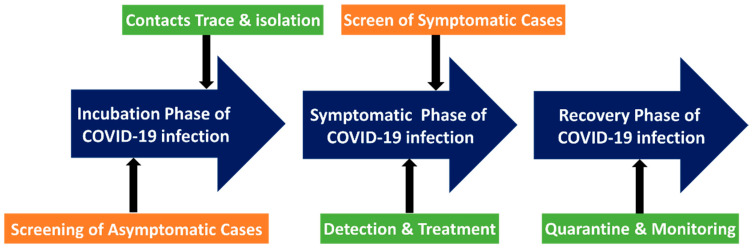
In order to screen the COVID-19 patients, diagnostic testing at point of care could be employed. The screening could happen at any time, from the incubation stage to the symptomatic stage. At various stages, various measures were implemented.

Due to its rapid transmission, the mass screening of SARS-CoV-2 is critical for containing its outbreak. In many countries, mass screening has become a common practice and an essential part of containment strategies [124]. Singapore implemented a thorough monitoring system in 2020 to find as many infections as possible and contain them on a personal level [124]. Furthermore, it was combined with community-based interventions based on the risk of transmission. This approach has been effective in limiting spread. Singapore’s COVID-19 monitoring aimed to detect as many infections as possible by employing conventional detection technologies. Methods for mass screening detection included serological tests, antigen assays, and polymerase chain reaction (PCR) tests. To expand the capacity for diagnostics, all public hospital laboratories in Singapore provide COVID-19 PCR testing. Serological tests were performed to investigate possible links between cases and clusters. PCR detection of respiratory tract specimens in the lab is the gold standard for the diagnosis of COVID-19. Furthermore, point-of-care methods and serological immunoassays are rapidly evolving. There are a growing number of screening techniques. However, several issues persist in many countries. Many wealthy countries have encountered difficulties with specimen collection and testing, slowing the expansion of testing capacity [125]. These difficulties can be significantly more severe in a resource-constrained setting. Due to pressing clinical and public health concerns, an extraordinary global effort is currently underway to improve the ability to detect SARS-CoV-2 infection. Several diagnostic tests were performed at various times on those with confirmed or suspected COVID-19 (Figure 8).

Imagine this idealized mass screening scenario: the SERS-based COVID-19 detection setup would be intended for use in-person at the location of patient care, which could include hospital laboratories, emergency rooms, intensive care units, outpatient clinics, and doctors’ offices [126]. An ill person with COVID-19 symptoms arrives at a hospital emergency room. In an ideal scenario, the doctor could swiftly and easily examine the sample (swabs, sputum, blood, urine, stool, etc.) in the examination room, which in turn should provide him with enough information to identify COVID-19. 

The mass screening might be utilized to check for asymptomatic infection during the SARS-CoV-2 virus’s incubation phase. Many factors must be considered when designing tests, such as cost, batch processing, throughput, turnaround time, the limit of detection, specificity testing, and assay sensitivity. It is critical to understand the acceptable diagnostic accuracy of the test [127]. Rapid and accurate SARS-CoV-2 diagnostics may aid in the development of clinical and public health plans to combat the COVID-19 pandemic. Generally, traditional screen tests including enzyme-linked immunosorbent assay (ELISA), polymerase chain reaction (PCR), and reverse transcription-PCR (RT-PCR), among others, provide gold-standard pandemic management solutions by enabling rapid and effective diagnosis SARS-CoV-2. However, such procedures cannot be used in the POC testing setup. Comparatively, SERS can provide quantitative data with excellent specificity, sensitivity, and multiplex detection capacity, and has previously been employed for POC settings [128]. 

**Figure 9:**
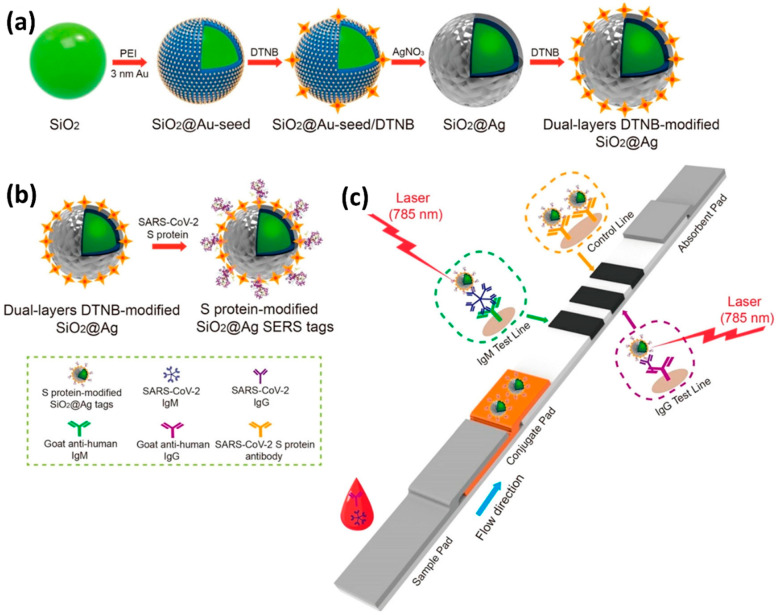
(**a**) Schematic of the dual-layer Raman reporter molecule 5,5′-dithiobis(2-nitrobenzoic acid (DTNB) modified SiO_2_-Ag Nanoparticles via LFIA. (**b**) SARS-CoV-2 S-protein-modified SiO_2_-Ag SERS probes preparation. (**c**) The SERS-LFIA strip’s operating concept for simultaneous high-sensitivity anti-SARS-CoV-2 IgM/IgG identification. Reproduced with permission from ref. [130]. Copyright 2021 Elsevier.

Liu et al. conducted a study that used the SERS method to identify COVID-19 at the point of care using a portable Raman instrument [129]. The authors employed Raman molecules functionalized two-layer silver-coated SiO_2_ NPs as SERS tags. The SERS tag was subsequently coupled with the SARS-CoV-2 virus’s s protein enabling concurrent diagnosis of IgM and IgG anti-bodies. Using a 785 nm excitation with 10.0 mW laser power for 1 s, Raman recordings were made on 19 COVID-positive samples and 49 COVID-negative samples. The results were obtained in the optimal time frame of 25 min, and the detection limit was 1 ng/mL of the S-protein antibody using a 240 nm SiO2-Ag nanoprobe, indicating high sensitivity and fast speed of the SERS probe. Figure 9 shows a schematic of the dual-layers of Raman reporter molecule 5,5′-dithiobis(2-nitrobenzoic acid (DTNB) modified silica-silver nanoparticles (SiO_2_-Ag) via Lateral flow immunoassay (LFIA) [130].

**Figure 10:**
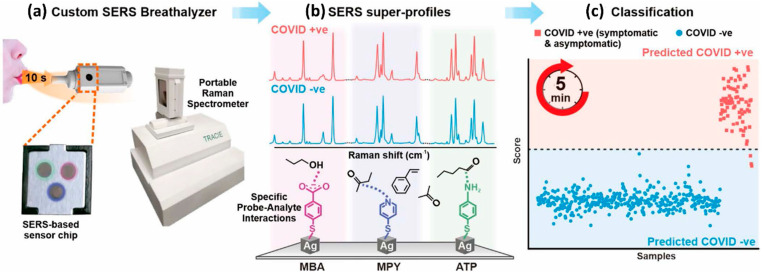
A flow-diagram of the SERS-based method for detecting COVID-positive patients utilizing volatile organic molecules in their breath (BVOCs). Reproduced with permission from ref. [131]. Copyright 2022 American Chemical Society.

Similarly, Shi Xuan Leong et al. [131] developed a handheld breathalyzer to identify COVID-19 patients within five minutes attaining >95 percent specificity and sensitivity across 501 subjects from a clinic case-control research study in Singapore as shown in Figure 10. Using the SERS-based breathalyzer, the authors recorded significant changes in vibrational signatures occurring from interactions between multiple molecular receptors and breath metabolites, and developed a classification algorithm for high-throughput spectrum studies based on the partial least squares discriminant analysis (PLSDA), which was integrated with portable Raman instruments to provide an instant result (within 5 min, and no sample preparation was required). The authors observed a strong relationship between COVID-19 patients and breath volatile organic compounds (BVOCs) as breath biomarkers, both through experimentation and simulation. Importantly, the authors showed that COVID-19 breath biomarkers spectrum variations are not reliant on COVID-19 symptoms and other possible extraneous variables including participants’ last meal time, tobacco smoking, gender, and age.

Table 3 shows a list of SERS techniques for the diagnosis of the corona virus. As depicted in this table, Zhang et al. [132] developed a “capture-quenching” strategy-based method to detect SARS-CoV-2 quickly without any RNA extraction step. The SERS probe was developed using a silver-nanorod array that was modified with the cellular receptor angiotensin converting enzyme 2 (ACE2). The SARS signal attenuation was found as a sign of SARS-CoV-2 presence based on either spectral variations or a red shift. The authors reported powerful SERS signals of ACE2 at 1527, 1447, 1189, 1089, 1051, and 1032 cm ^1^. The coupling of the SARS-CoV-2 spike protein’s receptor binding domain (RBD) to the SERS probe resulted in a shift and significantly reduced the SERS signal of most peaks. For spectral classification, the authors also employed multivariate analysis. 

Yang et al. [133] developed angiotensin converting enzyme 2 (ACE2)-modified Au “virus traps” as an ultra-sensitive SERS probe. The prepared probe specifically trapped and quickly detected SARS-CoV-2 in the polluted water with single virus level sensitivity. The prepared probe showed a six-fold increase in viral enrichment due to ACE2’s strong affinity for the Spike protein and “virus-traps” made of oblique gold nanoneedles. Furthermore, the author reported a nine-fold increase in Raman signal amplification due to multicomponent SERS effects. Moreover, machine-learning and classification algorithms were used to build the viral signal identification standard, resulting in an extremely low detection limit of 80 copies mL^−1^. 

Pramanik et al. [134] developed anti-spike antibody-coupled AuNPs as SERS probes for the quick detection of COVID-19 viral antigens using colorimetric change observation within 5 min. The SERS measurements were carried out using a portable Raman instrument. In the presence of the COVID-19 antigen, the AuNPs form clusters, changing color from pink to blue, allowing antigen determination by the naked eye at concentrations as low as 1 nanogram (ng) per mL. Importantly, the clustered AuNPs form “hot spots” that allowed the anti-spike antibody and 4-aminothiophenol coupled AuNPs to provide very strong SERS signal enhancement.

Zhang et al. [135] developed a rapid detection platform (1 to 2 min) based on SERS. The authors prepared the SERS probe by incorporating calcium ions and acetonitrile into the AgNPs’ reinforced substrate. The limit of detection was found to be 100 copies/test for H1N1 influenza, Human Adenovirus 3, and SARS-CoV-2, with excellent SNR values. Machine learning methods were also used to differentiate the three virus molecules using 1000 groups of each virus’s spectra subjectively. Acetonitrile was discovered to be an effective internal marker for controlling the signal strength of viral molecules in serum and saliva. The SERS signal strength was also used to quantify the viral concentration in saliva and serum, and the authors found a linear relationship. 

Similarly, Abdullah et al. [136] used saliva to detect SARS-CoV-2 proteins swiftly at extremely low concentrations. To simulate a real case situation, the authors used an extremely low concentration of 10^−9^ M SARS-CoV-2 s protein and SARS-CoV-2 receptor-binding domain (RBD) in saliva. The SERS substrate was composed of a gold- and silver-deposited silicon nanorod. The authors used the prepared SERS substrate to detect SARS-CoV-2 S and RBD proteins in saliva samples without further treatment and observed the linear response in a concentration-dependent study.

**Table 3:** Representative studies of the coronaviruses (COVID-19 and SARS CoV-2) by SERS.

Name of Virus	LoD/Virus Concentration	Laser (nm)	Strategy/ Type of Measurement	SERS Substrate	Ref.
SARS-CoV-2	-	-	Multivariate analysis	ACE2@SN-SERS substrate	[132]
COVID-19	153.3 pM, 230.37 pM	526, 558	LSPR	Silver nanodot	[137]
COVID-19	17.7 pM	785	-	Gold nanoparticles	[133]
COVID-19 Viral antigen	~4 pg/mL	-	-	Gold nanoparticles	[134]
SARS-CoV-2	$10^{2}$ vp/mL	785 nm	Multivariate analysis	AgNP substrate	[138]
SARS-CoV-2	100 PFU/test	633 nm	-	Ag@BCNPs (based on silver nanoparticles)	[135]
SARS-CoV-2 S	$10^{-9}$ M	632.8 nm	Concentration-dependent study	Silicon nanorod substrates	[136]

### 5.2. SERS-Based Detection of Influenza A(H1N1) Virus

Influenza viruses are among the leading causes of respiratory tract infection in humans, causing seasonal/endemic diseases. The influenza A virus of the H1N1 subtype has been recorded as a pandemic twice: in 1918, when the H1N1 strain arose as a zooanthroponosis infection of swine-origin (swine flu), and in 2003, when the H1N1 strain appeared as the Spanish flu [139,140]. The mortality of swine flu was projected to be 100–500 million during one year, with a case fatality rate (CFR) of 2.5% [141].

In April 2009, a new strain of the H1N1 swine flu surfaced in the United States, according to the Centers for Disease Control and Prevention (CDC) [142]. The new virus, known as A(H1N1) pdm09 or p(H1N1), killed around 575,400 people in one year, with a case fatality rate for symptomatic cases with medical attendance of roughly 0.05 percent. In combatting H1N1 influenza, the viral culture, quick antigen test, direct immune fluorescence (DFA), and real-time PCR were developed as laboratory diagnostic procedures for H1N1 influenza identification. Likewise, there is a large range of biosensors, such as SERS, that use antigens, enzymes, proteins, and DNA as biological components in combination with thermal, piezoelectric, optical, or electrochemical transducer elements for H1N1 influenza identification [143,144]. 

**Figure 11:**
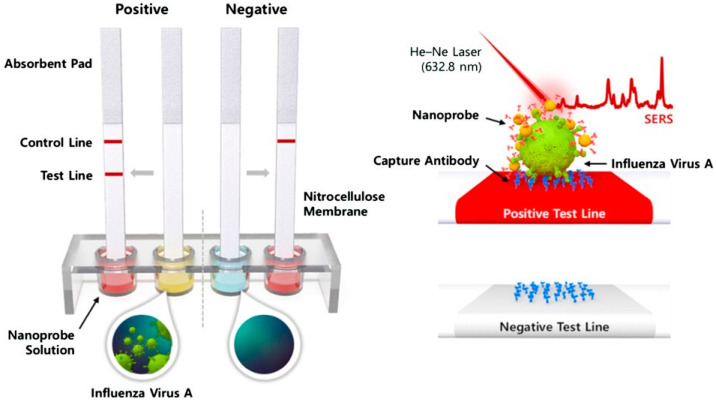
Schematic representation of the SERS-based LFA. (**a**) When the A(H1N1) was present in the sample solution, the SERS-virus complexes were formed and captured by the test line anti-bodies; excess SERS nanoprobes continued to flow and were captured by antibodies in the control line. In this scenario, the buildup of AuNPs causes both the control and test lines to turn red (**left**, positive). If the fluid contains no viruses, just the control line goes reddish (negative, **right**). (**b**) The associated SERS-virus nanoprobe complexes produced a SERS spectrum (**top**), but no signal was produced in the absence of the virus (**bottom**). Reproduced with permission from ref. [95]. Copyright 2016 John Wiley & Sons, Inc.

SERS has been used to identify and quantify different strains of A(H1N1) influenza. Particularly, identification studies were performed on solid substrates (refer to Table 4) without the use of linker molecules. In this approach, the analyte is brought into contact with the SERS substrate through the entrapment of the target in the interstices formed between metallic nanoparticles. Park et al. [95] described a SERS-based lateral flow assay for rapid on-site sensing of A(H1N1) influenza virus (Figure 11). The authors achieved precise detection of the influenza virus by recording distinctive SERS spectra of Raman-dyed tagged gold nanoparticles (AuNPs). The SERS spectra were collected in the test lines of the lateral flow assay device by replacing gold nanoparticles in the standard fast kits with SERS nanoprobes. A linear connection between the influenza virus A concentration and Raman signal strength was found in the range of $0-1.0\times 10^{6}$ PFU/mL with a limit of detection of $1.9\times 10^{4}$ PFU/mL.

### 5.3. SERS-Based Detection of A(H3N2)

Avian influenza is a highly infectious viral illness that mostly affects birds and poultry. The virus has scattered from Asia to Africa and Europe since early 2000, infecting large numbers of chickens and causing major financial harm to the rural economy. Human transmission and fatalities have also been reported [145].

**Figure 12:**
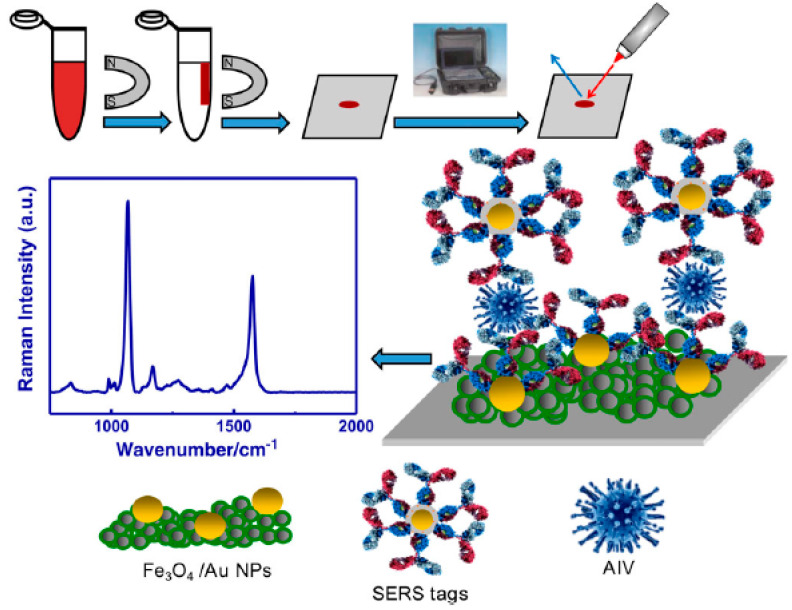
Illustration of an avian virus detection magnetic immunoassay based on surface-enhanced Raman scattering (SERS). Reproduced with permission from ref. [150]. Copyright 2017 Elsevier.

In combatting A(H3N2) virus, a PCR assay is the primary laboratory test for detecting the A(H3N2) virus [146]. Alternative detection routes include SERS, electrochemistry [147], colorimetry [148], and surface plasmon resonance (SPR) imaging [149]. Sun et al. studied A(H3N2) detection using a point-of-care SERS device with a complex built of two kinds of multilayer nanoparticles: (i) Gold nanoparticles modified with A(H3N2) immunoglobin G (IgG) and 4-mercaptobenzoic acid (4-MBA); (ii) Fe₂O₃ nanoparticles functionalized with influenza A immunoglobulin G and coated with gold nanoparticles. When nanoparticles in (i) and (ii) were combined with the subtype H3N2 of A(H3N2), the complex obtained, as shown in Figure 12, exhibited both magnetism (Fe₂O₃ nanoparticles) and Raman reporter (4MBA) properties [150]. As a result, the complex was isolated from the sample environment using a magnetic force and purified before taking Raman recordings. The authors also presented a comparison of SERS to various identification techniques, highlighting how SERS delivered a similar detection limit with a quicker analysis time compared to PCR-related detection techniques.

In another study, Moon et al. utilized immunoreaction to capture the influenza A/CA/07/2009 (pH1N1) virus on a sensing platform aided by the SERS antibody tag [151]. The SERS antibody probes were simply made by combining the gold nanoparticles binding peptide (GBP)—protein G—and the antibody in the absence of any difficult chemical or biological interactions. The SERS measurements detected the virus at lower concentrations of $4\times 10^{3}$ TCID per mL after silver amplification of the tag. The authors additionally presented a comparative study of SERS to various identification techniques, highlighting how SERS produced a similar limit of detection with less evaluation time compared to polymerase chain reaction-based (PCR) detection techniques. Table 4 shows a list of SERS techniques for the diagnosis of influenza.

Eom et al. [152] developed functional gold nanoparticles (AuNPs) capable of detecting oseltamivir-resistant (prpH1N1/H275Y) viruses in both SERS and naked-eye experiments. The functional AuNPs were produced by simultaneously functionalizing the surfaces of AuNPs with oseltamivir hexylthiol (OHT) and malachite green isothiocyanate (MGITC). The authors chose OHD due to its excellent binding affinity for the pH1N1/H275Y mutant virus. In its neutral state, the functional AuNPs were red but turned to purple in the presence of the prpH1N1/H275Y mutant virus. Apart from this calorimetric detection, the authors also detected the virus quantitively with a LoD value of 10 PFU based on the SERS signal intensity of MGITC. In another study, Wang C. et al. synthesized novel Ag-coated Fe_3_O_4_ magnetic nanoparticles (Fe_3_O_4_ @Ag) [153] as the SERS-based probe for simultaneous detection of influenza A H1N1 virus and human adenovirus (HAdV). The probes were coupled with target virus-capture antibodies and Raman reporters. The detection limit of the magnetic SERS strips for influenza H1N1 and human adenovirus (HAdV) were 50 pfu/mL and 10 pfu/mL, respectively [153]. Focused Ion Beam was employed by Sivashanmugan, K. et al. [154] to develop ordered Ag/Au nanorod arrays with a varying thickness of Au and Ag layers as the SERS probe for the detection of the influenza A virus strain. The number of repetitive layers of nanorod arrays enhanced the SERS signal by producing the electromagnetic effect at the Au surface. The limit of detection for A/Philippine/2/82 (H3N2)), A/England/12/64 (H2N2), and (A/WSN/33 (H1N1) virus strains was found to be 106 PFU/mL [154]. 

Another study reported inverted triangular Au nano-cavities as a substrate for qualitative encephalomyocarditis and influenza virus detection. The authors found that viruses can be differentiated based on amino acids on their surface by inducing an electro-magnetic influence through a customized substrate [155]. The limit of detection for adenovirus was 107 PFU/mL, while influenza virus was detected at a concentration of 104 PFU/mL [155]. The authors also stated that the Au nano-cavity-based substrate is suitable for qualitative virus determination and is largely independent of virus concentration.

**Table 4:** Representative studies of the direct and indirect identification of the A(H1N1) influenza virus by surface-enhanced Raman spectroscopy (SERS).

Name of Virus	LoD/Virus Concentration	Laser (nm)	Strategy/Type of Measurement	SERS Substrate	Identification	Ref.
A/CA/07/2009 (pH1N1)	$4\times 10^{3}$ (TCID/mL)	632	Immunoassay	AuNps–Ag–protein G–glass substrate	Indirect	[151]
(pH1N1)/H275Y mutant	10 PFU	633	Functional nanoparticles	-	Indirect	[152]
A/FM/1/86 (H1N1)	50 PFU/mL	785	Immunoassay	pAb–LFIA strip of nitrocellulose molecules	Indirect	[153]
A/WSN/33 (H1N1)	$1\times 10^{6}$	633	Wet	Au/Ag multilayered nanorod arrays onto Single-Crystal Silicon	Direct	[154]
A/WSN/33 (H1N1)	$1\times 10^{4}$	633	Dry	Au substrate on Single-Crystal Silicon	Direct	[155]
A/Taiwan/N39/06 (H1N1)	$1\times 10^{6}$	633	Wet	Au nanorods onto Single-Crystal Silicon	Direct	[156]
A/WSN/33 (H1N1)	$1\times 10^{4}$	633	Wet	Au nanorods onto Single-Crystal Silicon	Direct	[157]
A/California/04/2009 (H1N1)	__	785	Dry	Aggregates of spherical AuNPs on the cover of glass	Direct	[158]

A focused ion beam (FIB) approach was used in a different study to create gold nano-rod patterns with varying spacings accurately as a SERS-active substrate [156]. Gold nano-rods were arranged with a separation (*D*_R_) to detect viruses with the size of (*D*_T_); three viral strains (i.e., adenovirus, encephalomyocarditis, and influenza virus) were tested. A strong interaction between the target virus and the electromagnetic field was achieved when the *D*_R_/*D*_T_ ratio became 1 [156]. By changing the geometry, dimension, and spacing of Au micro/nanostructures, the role of SERS on the focused ion beam (FIB) fabricated Au micro/nanostructures were examined in another study. The SERS process in these micro-/nanostructures was investigated through the use of low concentration R6G as the target molecule and the 633 nm laser for excitation [157]. After evaluating the effectiveness of the SERS mechanism, an optimized Au nanostructure, obtained from FIB, was utilized to detect low-concentration influenza [158]. Likewise, a different study explored influenza virus identification using SERS, with the overall goal of ensuring rapid and accurate identification of virus-related diseases. The authors reported a non-replicating pseudo type virus with influenza virus elements exhibiting higher SERS intensity on AuNPs that was distinct from the pseudo type with a non-influenza virus element. Additionally, Raman peaks from viruses with surface elements of influenza virus, A/California/04/2009, differed from those produced by viruses with elements of standard lab influenza [158].

## 6. Future Perspectives

As evident in the coronavirus disease (COVID-19) pandemic, virus propagation is a global threat that necessitates the continuous development of new analyses and techniques for virus detection. Since most viruses can be easily transmitted through contact with infected areas, it is more crucial to develop rapid and accurate tools for virus detection at the point of care to minimize pandemic outbreaks. One promising technique for virus detection at the point of care is SERS; several viruses have been identified using the miniaturized SERS instrument. SERS spectra bands can be utilized to detect viral molecular fingerprints. Currently, to categorize and analyze SERS spectra bands with small differences, statistical algorithms, including machine learning methods, are used. To quantify the concentration of the virus, the calibration curve is generated using a set of solutions with known concentrations of the virus such that the individual SERS peak linearly increases as a function of virus concentrations. Instead of analyzing an individual SERS peak, an entire SERS spectrum can also be used for virus quantification by employing machine learning methods. Despite the current advancement of SERS-based viral detection, in the future, SERS-based point-of-care systems must improve automation, miniaturization, and portability. Over the last decade, Raman instruments dramatically decreased in size and cost and are now widely available; however, more work is needed to improve the convenience of use and shorten the time between data collection and tangible results, as well as to develop a reasonably priced, therapeutically reliable, and rapidly deployable system. In reality, multiple projects are being conducted in this direction, and we anticipate that SERS will attain its full potential in the near future.

## 7. Conclusions

This review looked at SERS strategies in identifying a variety of respiratory viruses, such as influenza A virus subtypes such as H1N1 and the new coronavirus SARS-CoV-2, with a focus on point-of-care diagnosis using portable Raman instruments. Our study found that the use of portable Raman instruments has increased significantly in the last 5–10 years, owing to the appealing prospect of performing in situ analysis with simple equipment, versatility, faster turnover, simpler protocols, and lower costs. We also compared a number of commercially available handheld Raman instruments in terms of laser wavelength, power, spectroscopy geometry, and detector types. An insightful comparison of handheld Raman devices and portable Raman devices was also investigated. A discussion of the SERS substrate for point-of-care applications, with a focus on virus detection, is also provided. According to the data we gathered, SERS-based techniques for virus identification are considered fast, accurate, and practical, and should continue to attract more attention. Instrumentation and commercialization advancements are expected to expand the use of SERS as a quick and cost-effective analytical method for virus detection.

## Figures and Tables

**Table 2:** A comparative assessment of the bench-top and portable Raman device manufactured by Thermo Scientific Company. Table adapted with permission from [84]. Copyright 2014 Elsevier.

Merits/Demerits	Desktop	Handheld
Signal Variation	Relatively high	Relatively low
On-site detection	Not portable	Portable
Scanning Range	Broad	Narrow
Sensitivity	Relatively High	Relatively low
Adjustability	Adjustable	Fixed
Price ($)	Expensive	Inexpensive
Intelligence	Required manual analysis	Intelligent
**Parameter Comparison**		
Size (cm^3^)	(97 × 69 × 61)	(30 × 15 × 7.6)
Weight (kg)	56.7	1.7
Power (mW)	0–24 (adjustable)	300 or less
Estimated resolution (cm^−1^)	4.7–8.7	7.0–10.5
Wavenumber range (cm^−1^)	50–3400	250–1875
Estimated spot size (mm)	1000–3000 mm (adjustable)	1–2 mm
Exposure Time (s)	Adjusted as required	~40
Laser	780 (could be 632 and 532)	785 ± 5

## Data Availability

The data presented in this study are available from the corresponding authors upon request.
